# Prenatal administration of IL-1Ra attenuate the neurodevelopmental impacts following non-pathogenic inflammation during pregnancy

**DOI:** 10.1038/s41598-021-02927-3

**Published:** 2021-12-03

**Authors:** Marie-Eve Brien, Katia Hughes, Sylvie Girard

**Affiliations:** 1 Ste-Justine Hospital Research Center, Montréal, QC Canada; 2 Department of Microbiology, Infectiology and Immunology, Université de Montréal, Montréal, QC Canada; 3 Department of Obstetrics and Gynecology, Université de Montréal, Montréal, QC Canada; 4 Department of Obstetrics and Gynecology, Department of Immunology, Mayo Clinic, 222, 3rd Ave SW, Rochester, MN 55905 USA

**Keywords:** Intrauterine growth, Acute inflammation

## Abstract

Prenatal inflammation negatively affects placental function, subsequently altering fetal development. Pathogen-associated molecular patterns (PAMPs) are used to mimics infections in preclinical models but rarely detected during pregnancy. Our group previously developed an animal model of prenatal exposure to uric acid (endogenous mediator), leading to growth restriction alongside IL-1-driven placental inflammation (Brien et al. in J Immunol 198(1):443–451, 2017). Unlike PAMPs, the postnatal impact of prenatal non-pathogenic inflammation is still poorly understood. Therefore, we investigated the effects of prenatal uric acid exposure on postnatal neurodevelopment and the therapeutic potential of the IL-1 receptor antagonist; IL-1Ra. Uric acid induced growth restriction and placental inflammation, which IL-1Ra protected against. Postnatal evaluation of both structural and functional aspects of the brain revealed developmental changes. Both astrogliosis and microgliosis were observed in the hippocampus and white matter at postnatal day (PND)7 with IL-1Ra being protective. Decreased myelin density was observed at PND21, and reduced amount of neuronal precursor cells was observed in the Dentate Gyrus at PND35. Functionally, motor impairments were observed as evaluated with the increased time to fully turn upward (180 degrees) on the inclined plane and the pups were weaker on the grip strength test. Prenatal exposure to sterile inflammation, mimicking most clinical situation, induced growth restriction with negative impact on neurodevelopment. Targeted anti-inflammatory intervention prenatally could offer a strategy to protect brain development during pregnancy.

## Introduction

Inflammation during pregnancy has important adverse effects on newborns, through increased risk of pregnancy complications, as well as elevated incidence of mortality and neurodevelopmental diseases in survivors^1–6^. These have major long-term health impacts on the children, without forgetting the emotional burden on affected families^7^. Prenatal inflammation has been shown to directly alter placental function, subsequently affecting newborn neurodevelopment, particularly motor and cognitive functions^1, 8^. Several preclinical studies have shown the causal link between inflammation of pathogenic origin in pregnancy and brain damage^1, 2, 5, 9–14^. Furthermore, it has been demonstrated that targeting the placenta was key in order to protect postnatal brain development against the negative impact of prenatal exposure to inflammation^1, 8, 15^. In case of fetal growth restriction previous studies, using pathogen-induced inflammation have observed decreased pup’ survival and, in surviving pups, the phenotype observed is often more severe than what is reported in human^13, 16^. In human, term FGR is often associated with a milder neurodevelopmental presentation than in neonates developing FGR early in pregnancy and in which case it is often combined to preeclampsia or prematurity (iatrogenic in most cases due to failure to thrive). In the latter, gestational age is the major determinant for fetal status at delivery especially in terms of neurodevelopment^17–22^.

It is difficult to extrapolate data from studies using pathogen-derived inflammation to the clinical setting since pathogens are rarely detected in complicated pregnancies, although inflammatory mediators are still present^9^. Alarmins, or damaged-associated molecular patterns (DAMPs), are another cause of inflammation, increasingly associated with pathological pregnancies^23–32^. However, the mechanisms linking inflammation particularly of sterile or non-pathogenic origin (i.e. DAMPs-induced), and the negative impacts on neurodevelopment are still unknown which makes it difficult to develop new therapeutic strategies.

Our group developed an animal model of non-pathogenic inflammation during pregnancy, induced by uric acid crystals^33^. Elevated uric acid levels have been shown to be associated with complicated pregnancies such as preeclampsia^34–36^. In this model, uric acid crystals were injected in the late gestation period in rats, leading to placental inflammation with increased levels of cytokines (e.g. IL-1β, IL-6, TNFα) and immune cells infiltration within the placenta. We also observed that uric acid injection at the end of the pregnancy induced fetal growth restriction (FGR)^33^.

Following up from this work, our objective was to investigate the postnatal impact of prenatal exposure to sterile or non-pathogenic inflammation induced by uric acid, particularly on brain development. Since we previously showed that the placental impact of in utero exposure to uric acid was mediated mainly through the IL-1 pathway as seen by reduced percentage of M30+ apoptotic cells and increased syncytialization with treatment of UA and IL-1 blockers^33^, we also tested the therapeutic potential of prenatal anti-inflammatory treatment through administration of the IL-1 receptor antagonist (IL-1Ra).

## Results

### Placental inflammation induced by uric acid exposure during pregnancy was prevented by prenatal IL-1Ra treatment

**Figure 1:**
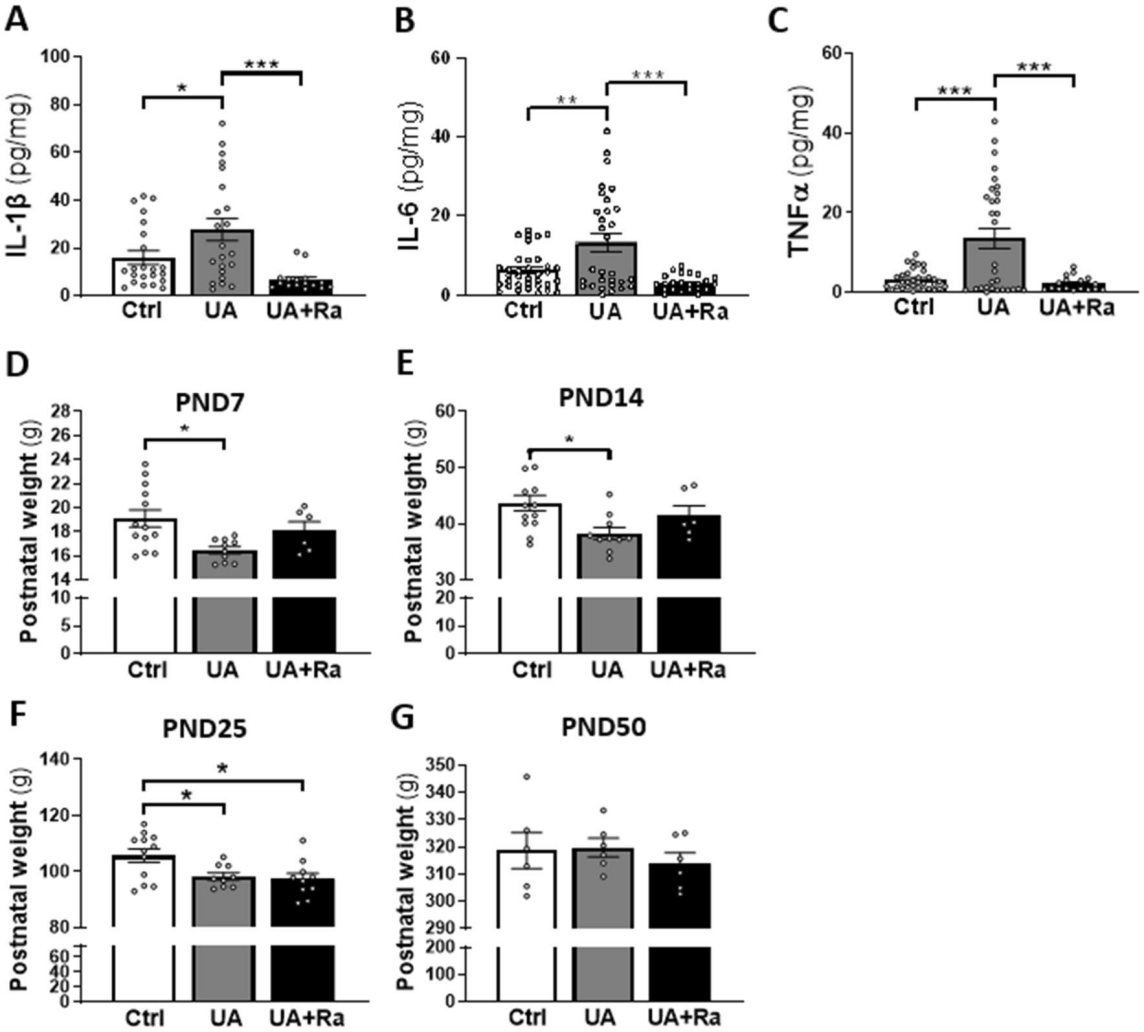
Placental inflammation and growth restriction induced by uric acid exposure during pregnancy. Sterile inflammation exposure led to increased placental inflammation and anti-inflammatory intervention decreased levels of IL-1β (**A**), IL-6 (**B**) and TNF-α (**C**) proteins at birth. N = 6–12 dams (i.e. Ctrl = 12; UA = 12; UA + Ra = 6), 3 placentas/dams. Quantification of postnatal weight at PND7 (**D**), PND14 (**E**), PND25 (**F**) and PND50 (**G**) after exposure to sterile inflammation. Treatment with UA led to decreased weight maintained in the early postnatal period. N = 6–10 litters (i.e. PBS = 8; IL-1Ra = 6; UA = 10; UA + Ra = 6) at PND7, 14 and 21 and N = 6 litters in each groups at PND50. *p < 0.05; **p < 0.01; ***p < 0.001; Results presented as mean ± SEM. Statistical analysis by one-way ANOVA and Tukey’s multiple comparisons test with with GraphPad Prism 9.2.0 (GraphPad Software, CA); www.graphpad.com.

Placental inflammation was observed at birth after UA exposure, as we already reported^33^, with increased IL-1β, IL-6 and TNF-α proteins, and those proteins were significantly decreased by prenatal IL-1Ra administration (Fig. 1A–C). Prenatal exposure to UA induced sustained postnatal growth restriction which was significant on PND7, PND14 and PND25 but the difference was lost at PND50 (Fig. 1D–G). Treatment with IL-1Ra did not protect against weight loss (Fig. 1).

### Structural alterations of the developing brain following prenatal exposure to non-pathogenic inflammation and partial protection by IL-1Ra treatment

**Figure 2:**
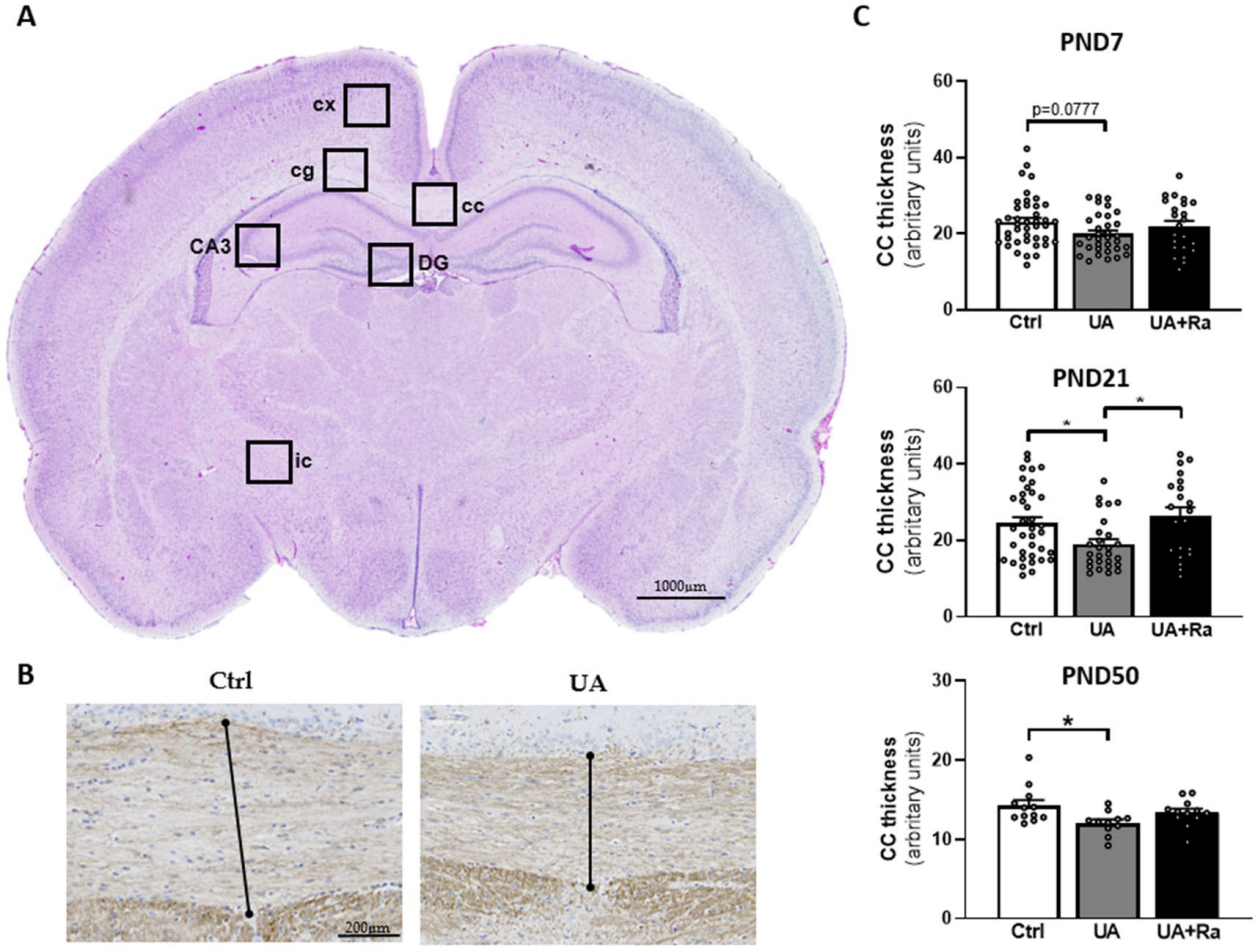
Morphological brain alteration following uric acid exposure. Representative image of HE staining of the brain regions studied (**A**). Representative image of MBP staining at PND21 to measure the corpus callosum thickness (**B**) and quantification at different postnatal time (**C**). *cc* corpus callosum, *cg* cingulum, *cx* cortex, *DG* hippocampus-dentate gyrus, *3-CA* 3 hippocampus-cornus amonis, *ic* internal capsule. N = 8–16 litters (i.e. PBS = 11; IL-1Ra = 8; UA = 16; UA + Ra = 12). *p < 0.05; **p < 0.01; ***p < 0.001. Results presented as mean ± SEM. Statistical analysis by one-way ANOVA with Tukey’s multiple comparisons test with GraphPad Prism 9.2.0 (GraphPad Software, CA); www.graphpad.com.

Histological analysis was performed on six regions of the brain in both the white and grey matter (see Fig. 2A) and at four different time points during development (see “Methods” section for further details). Pups exposed in-utero to UA had decreased corpus callosum thickness at PND7, 21 and 50 (Fig. 2B,C). Prenatal IL-1Ra was partially protective since corpus callosum thickness returned to control levels at PND21 (p < 0.05).

#### Micro- and astrogliosis following prenatal inflammation

**Figure 3:**
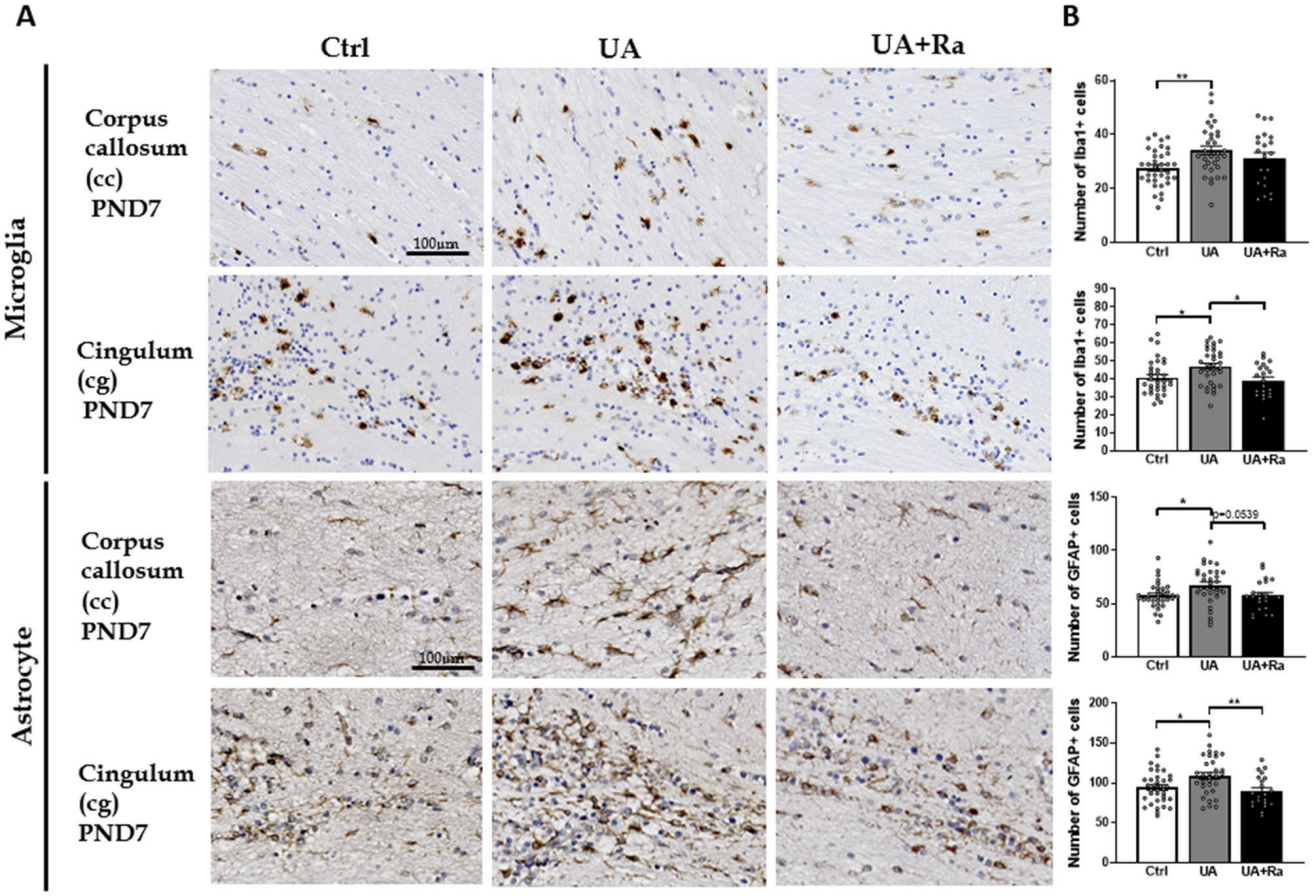
Histological analysis of Iba-1 and GFAP positive cell in the white matter following uric acid alone or with IL-1Ra prenatal treatment. Injection of uric acid during gestation increased the number of microglial cell and astrocytes in the corpus callosum and in the cingulum at PND7. Prenatal treatment with IL-1Ra reduced the number of microglia and astrocytes to a similar level than in the control group. Representative image shown in (**A**) and quantification in (**B**). N = 8–16 litters (i.e. PBS = 11; IL-1Ra = 8; UA = 16; UA + Ra = 12). *p < 0.05; **p < 0.01; ***p < 0.001 Results presented as mean ± SEM. Statistical analysis by one-way ANOVA with Tukey’s multiple comparisons test with GraphPad Prism 9.2.0 (GraphPad Software, CA); www.graphpad.com.

Prenatal exposure to inflammation led to microgliosis and astrogliosis, as seen by increased number of Iba1+ and GFAP+ cell, in the white matter (both corpus callosum (cc) and cingulum (cg)) at PND7 (Fig. 3). Treatment with IL-1Ra was protective against microgliosis and astrogliosis in the cingulum (Fig. 3).

**Figure 4:**
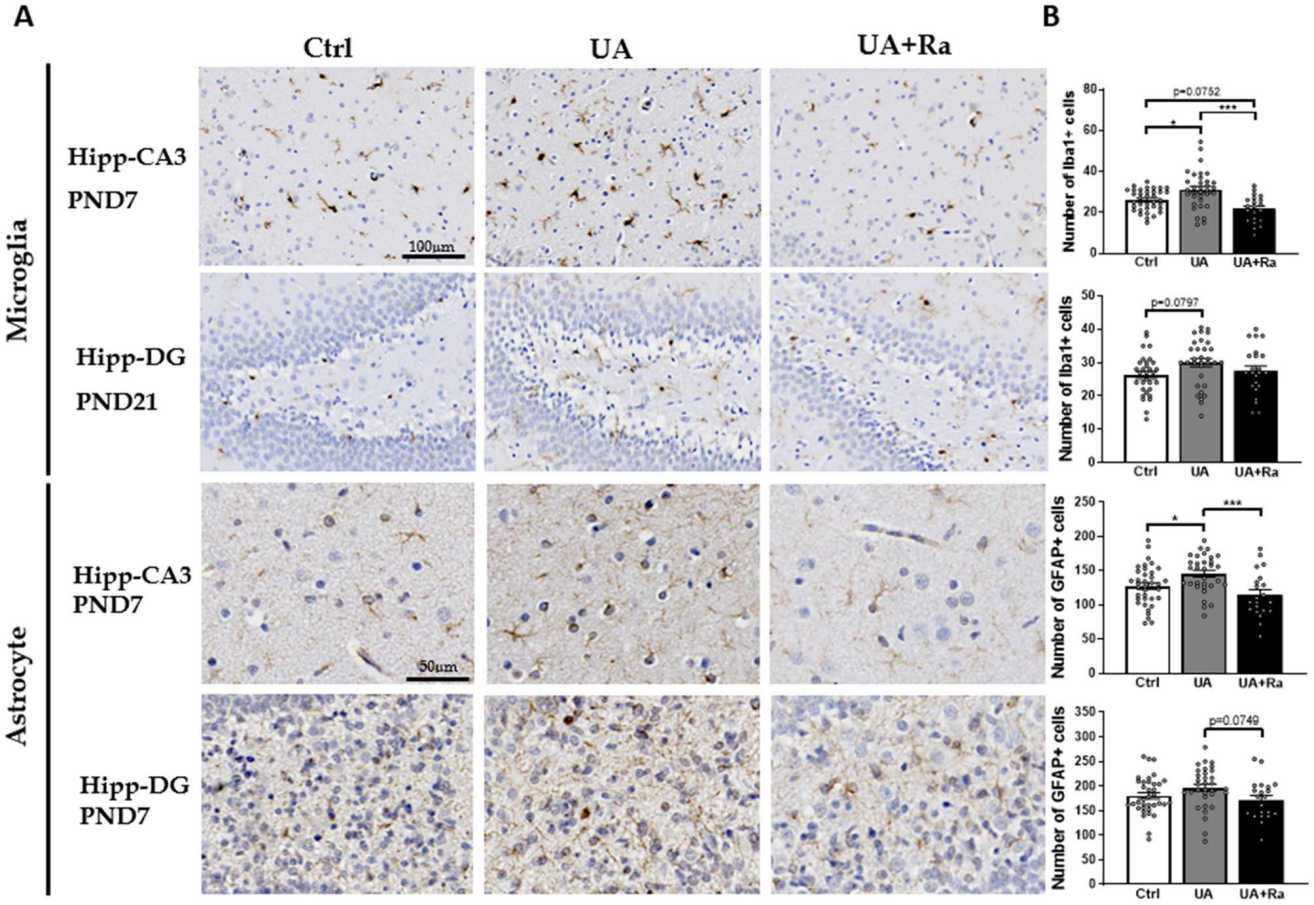
Histological analysis of Iba-1 and GFAP positive cell in the hippocampus following uric acid alone or with IL-1Ra prenatal treatment at PND7. Injection of uric acid during gestation increased the number of microglia and astrocytes in the hippocampus (CA3) at PND7. Prenatal treatment with IL-1Ra prevented the microgliosis and astrogliosis observed at PND7. Representative image shown in (**A**) and quantification in (**B**). N = 8–16 litters (i.e. PBS = 11; IL-1Ra = 8; UA = 16; UA + Ra = 12). *p < 0.05; ***p < 0.001 Results presented as mean ± SEM. Statistical analysis by one-way ANOVA with Tukey’s multiple comparisons test with GraphPad Prism 9.2.0 (GraphPad Software, CA); www.graphpad.com.

In the grey matter, microgliosis was observed in the hippocampus (CA3) at PND7 and tended to be still observed at PND21 in the dentate gyrus (DG) (Fig. 4). Astrogliosis was observed in the hippocampus at PND7 (Fig. 4). Prenatal treatment with IL-1Ra prevented microgliosis and astrogliosis in part of the hippocampus (i.e. CA3) at PND7 (Fig. 4). At later developmental stages (i.e. PND35 and PND50), no difference was observed between the groups for both micro- and astro-glial activation (data not shown).

#### Altered myelination and decreased neuronal precursor cells following prenatal inflammation

**Figure 5:**
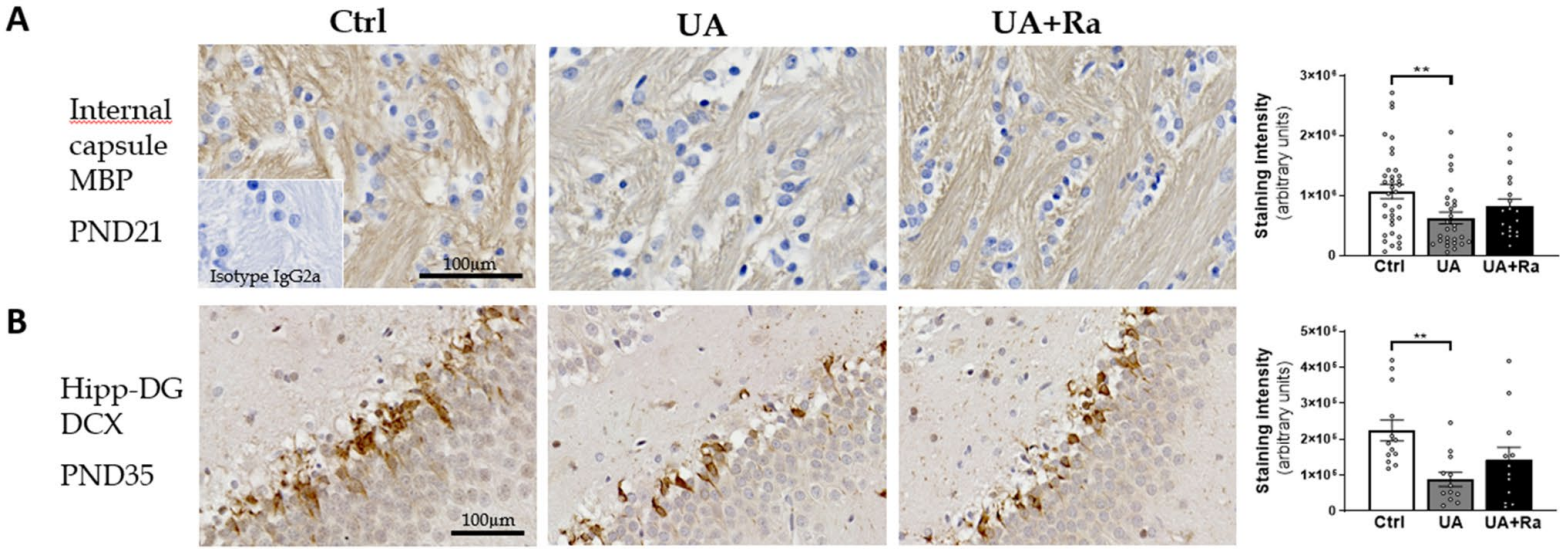
Myelin and neuronal precursor cell histological analysis following uric acid alone or with IL-1Ra prenatal treatment. Injection of uric acid during gestation decreased the myelin staining intensity in the internal capsule at PND21 (**A**) and decreased doublecortin staining in the hippocampus-DG at PND35 as opposed to the control group (**B**). Prenatal treatment with IL-1Ra did not statistically preserve these regions. N = 8–16 litters (i.e. PBS = 11; IL-1Ra = 8; UA = 16; UA + Ra = 12) for MBP and N = 6 litters/group for DCX. **p < 0.01. Results presented as mean ± SEM. Statistical analysis by one-way ANOVA with Tukey’s multiple comparisons test with GraphPad Prism 9.2.0 (GraphPad Software, CA); www.graphpad.com.

Decreased myelin staining intensity was observed at PND21 in the internal capsule of pups exposed prenatally to UA (Fig. 5A) but the difference was lost at PND50 (data not shown). Furthermore, the staining intensity for doublecortin (DCX), a marker of neuronal precursor cells, was decreased in the hippocampus of the pups exposed in-utero to UA at PND35 (Fig. 5B). Prenatal treatment with IL-1Ra was not protective against either myelin nor DCX cells loss (Fig. 5). The number of mature neurons, as detected by the NeuN antibody, was not changed by prenatal UA exposure (data not shown).

### Prenatal uric acid exposure induced motor impairments

**Figure 6:**
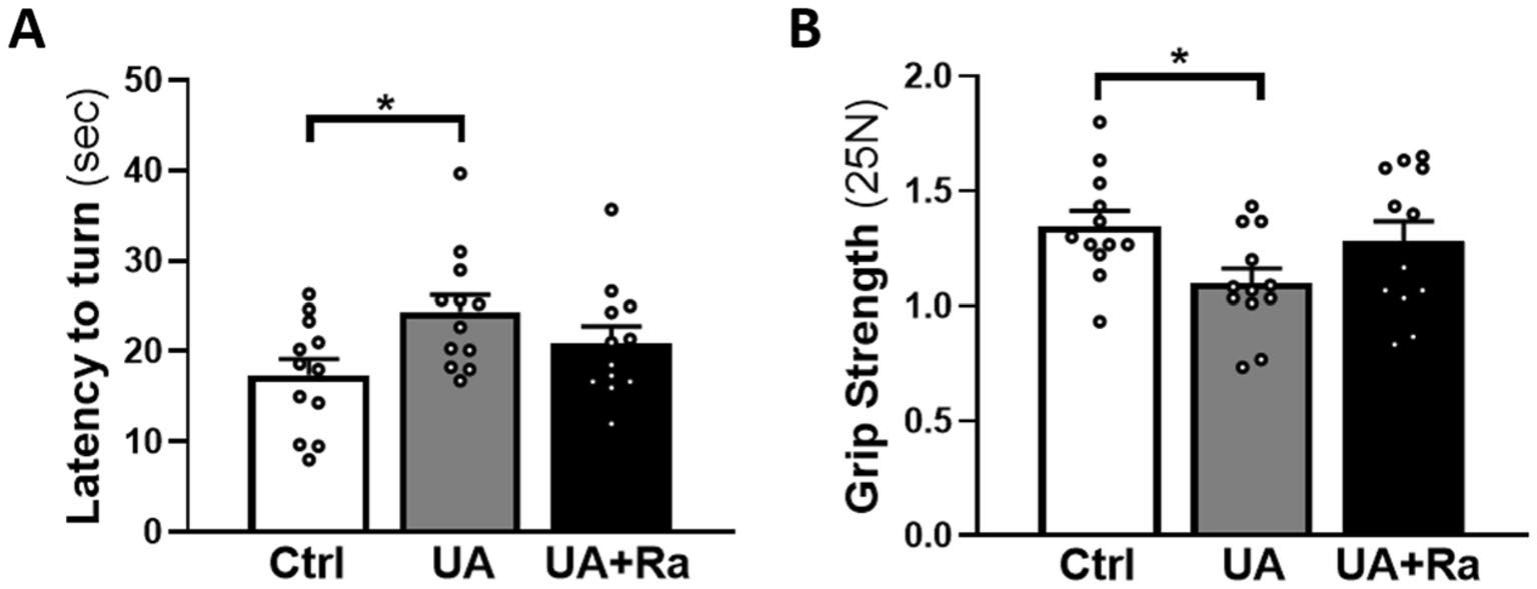
Impaired motricity following UA exposure during gestation. At PND7, prenatal inflammation induced a delay in turnaround time in the inclined plane in pups exposed to uric acid (**A**). At PND15, pups exposed in-utero to uric acid were weaker as evaluated by the grip strength test (**B**). N = 6 litters/group. *p < 0.05 Results presented as mean ± SEM. Statistical analysis by one-way ANOVA with Tukey’s multiple comparisons test with GraphPad Prism 9.2.0 (GraphPad Software, CA); www.graphpad.com.

Functional assessment of the pups revealed that prenatal UA exposure led primarily to motor impairments. Pups exposed prenatally to UA needed more time to fully turn upward (180 degrees) on the inclined plane at PND7 (Fig. 6A; 24 s vs 17 s in Ctrl, p < 0.05) and were weaker on the grip strength test at PND15 (Fig. 6B; 1.10 25N vs 1.34 25N in Ctrl, p < 0.05, at PND15). Those parameters were not statistically improved by IL-1Ra prenatal treatment. The catwalk test revealed a tendency to decreased regulatory index in UA exposed pups, meaning altered inter-paw coordination, the normal value being 100% (Supplementary Fig. S1A, p = 0.0687). Furthermore, the base of support (BOS) of the hind paws was decreased at PND16, as opposed to the Ctrl group, in pups exposed in-utero to UA (Supplementary Fig. S1B, p = 0.0555). All the other parameters such as cadence, print position, print area, stand, stride length, step cycle, stance, were unaltered. Furthermore, no difference was observed for the catwalk test at a later timepoint (i.e. PND36-38) across the groups.

On the other hand, tests for ultrasonic vocalisation (i.e. communication), exploratory behavior (i.e. open field test), social interaction, novel object recognition (i.e. short term memory) and anxiety (i.e. elevated plus maze) did not revealed any difference in pups exposed prenatally to UA-induced inflammation (data not shown).

## Discussion

In this work we used our previously published model of prenatal uric acid exposure leading to placental inflammation and associated FGR^33^, to investigate the impact on pup’s neurodevelopment as well as the therapeutic potential of prenatal anti-inflammatory treatment. For the latter, the IL-1 pathway was targeted, using the IL-1 receptor antagonist; IL-1Ra. We showed that prenatal exposure to non-infectious inflammation, induced by uric acid, led to postnatal growth restriction and induced both structural and functional alterations during pups postnatal development. Microgliosis and astrogliosis were observed transiently in both white and grey matter at PND7 alongside with motricity delayed as evaluated by increased time to turn upside on the inclined plane and weaker pups in the grip strength test. Prenatal treatment with IL-1Ra, decreased placental inflammation but had no effect of UA-induced postnatal growth restriction. IL-1Ra treatment also prevented against some structural brain damage (mostly against microgliosis and astrogliosis at PND7).

In the prenatal part of this work, we observed that concomitant administration of IL-1Ra with UA protected against placental inflammation, as seen by decreased levels of cytokines (IL-6 and TNF-α). This is in line with previously published studies using a pathogenic stimulus prenatally, namely LPS, in which case IL-1Ra administration during pregnancy was shown to be protective through its mitigation of placental inflammation^1, 15^.

In the postnatal part of this work, we followed the pup’s development and weight gain after natural birth. No preterm delivery was observed, as opposed to other model of non-infectious inflammation using for example IL-1α or HMGB1^29, 30^. In the HMGB1 mice model only intra-amniotic, and not intraperitoneal, injection led to preterm birth^30^. In another model by Schwenkel et al., intra-amniotic HSP70 injection induced low rate of preterm birth but important neonatal complications were observed^28^. In a model of fetal DNA administration during pregnancy, fetal resorption and preterm deliveries occurred^31^. These models of prenatal inflammation induced by DAMPs showcase the wide range of effects that can be observed during pregnancy. In all those models, the timing, mode of injection, DAMPs used, and species (mice vs rats) could explain the differences observed. Also, in all those models except the one using HSP70, there is no detailed follow up of the pups postnatally. In our model, we observed growth restriction but also that the lower weight persisted until PND25, which is in contrast with the LPS model in which pups gain back the weight rapidly after delivery^16^. Only one other model of prenatal inflammation presented growth restriction at birth which was still observed at PND40^5^. This model used prenatal administration of live bacteria (group B streptococcus), as compared to all other models mentioned above in which only PAMPs were administered.

We investigated both structural and functional changes at several time points during postnatal development following in-utero inflammation. We observed alteration of the corpus callosum with decreased thickness in the prenatal UA group. This is in agreement with what was observed in other models of prenatal inflammation^4, 37^. Furthermore, in our model, we observed microglial and astroglial activation similarly to what was reported in two reviews of PAMPs-induced prenatal inflammation models^6, 13^. Although the source of inflammation (i.e. DAMP vs PAMP) was different in the current study, there were still similarities in the lesions observed^1, 16, 38^. In preclinical models of pathogens induced inflammation (mainly using LPS), reduced litter size is observed with important fetal loss and FGR and white matter injury was predominant in surviving newborns^13, 16^. Interestingly, our current work showed only moderate white matter damage localised to the corpus callosum. Unlike previous work which used pathogens as initiator of inflammation, we focused on endogenous inflammation using the alarmin uric acid. In our study, the FGR is induced in late gestation without any decreased in litter size and no preterm delivery. This could explain in part why our results are not as striking as the one using PAMPs such as LPS, or mechanical ligation (reviewed elsewhere^20^). In an FGR model in sheep, by Alves de Alencar Rocha et al., early vs late-onset FGR were associated with different types of white matter injuries and inflammation with more widespread results in early-onset^39^. In another model of FGR in rats, Pham et al. reported white matter deficits, however this model used inhaled nitric oxide from G5 to 19 which leads to early FGR^40^. In several model using unilateral artery ligation from G19 to G22 in rats, different studies reported different outcome such as altered hippocampal astrocytes, transient astrogliosis, hypomyelinisation, locomotor, strength and coordination deficit with only some aspect persisting through adulthood^20, 41–44^. Those models are very useful to mimic FGR but they are mechanical ligation which is different to the phenotypic FGR in human.

In terms of functional impairments, motricity was primarily affected as evaluated by the inclined planed and grip strength tests. This is in accordance, although to a lower extent, with what was reported for LPS-induced prenatal inflammation^16^. Using the catwalk test, we observed a tendency of decreased regulatory index and base of support of the hind paws which has been shown to be altered in model of spinal injuries^45, 46^. In contrast, in models of PAMPs-induced prenatal inflammation (ex. LPS, GBS, poly I:C), other types of behavioural impairments were reported such as social interaction, communication and anxiety-like phenotype^4, 16^. However in our model, these phenotypes were not observed. These differences could be related to the timing of injection that was selected, late gestation (GD18-21) in the current model whereas earlier exposure was often used in poly I:C (GD9, 12.5 or 15) or LPS (GD10 or 15) models^13, 47–49^. Specific brain regions are susceptible to inflammation at precise period of gestation which could explain in part the model-specific differences observed. For example, overactivation of microglia in white matter during development, even if transient, has been shown to be detrimental^50, 51^. It would be highly relevant in future studies to address the phenotype of microglial cells and the long-term changes in their susceptibility to immune stimuli following prenatal exposure to UA-induced inflammation. This is important has the changes in morphology that is often associated with phenotypic change in microglial cells in adults are not as well defined in the developing brain^52^. Furthermore, the decrease in neuronal precursors cells we observed, even without change in the number of mature neurons (NeuN+), can still lead to increased susceptibility of the pups to challenges later in life. Importantly, we did not observe any changes in maternal weight or activity during pregnancy following administration of UA, unlike other models of pathogens-induced inflammation, further supporting differences in the impact on pups’ development.

IL-1Ra has already been used in several models of prenatal inflammation (all of pathogenic origins) and shown to be protective against placental inflammation and subsequently postnatal brain injury^1, 11, 12, 16, 53–55^. Our work using a novel model of sterile inflammation further supports the therapeutic potential of prenatal IL-1Ra. Since it has been shown that IL-1Ra does not cross the placenta, even in inflammatory cases in which the placental barrier might not be fully intact^10^, this anti-inflammatory option could be of high interest in order to target the placenta specifically without fetal transfer and risk of accumulation. This is truly important because a new study by Ganguly et al. has shown that postnatal inflammation in neonates can be impaired in babies that have been exposed directly to anti-inflammatory treatment^56^. Therefore therapeutic strategies that targets the placenta, without crossing to the fetal side, are of high interest. Our work further supports the beneficial use of IL-1Ra in models of inflammation of endogenous origin.

## Conclusions

Prenatal exposure to non-infectious inflammation, mimicking the most frequent clinical situation, has important negative impact on brain development. Prenatal anti-inflammatory intervention could be used to protect the developing brain by reducing placental inflammation.

## Methods

### Ethical approval

Animal work was approved by the Institutional Animal Care Committee at the Sainte-Justine Research Center and the Research Institute of the McGill University Health Centre (protocol: 2020-2676) and in line with the guidelines of the Canadian Council of Animal Care (CCAC) and the ARRIVE guidelines.

### Animal model

**Figure 7:**
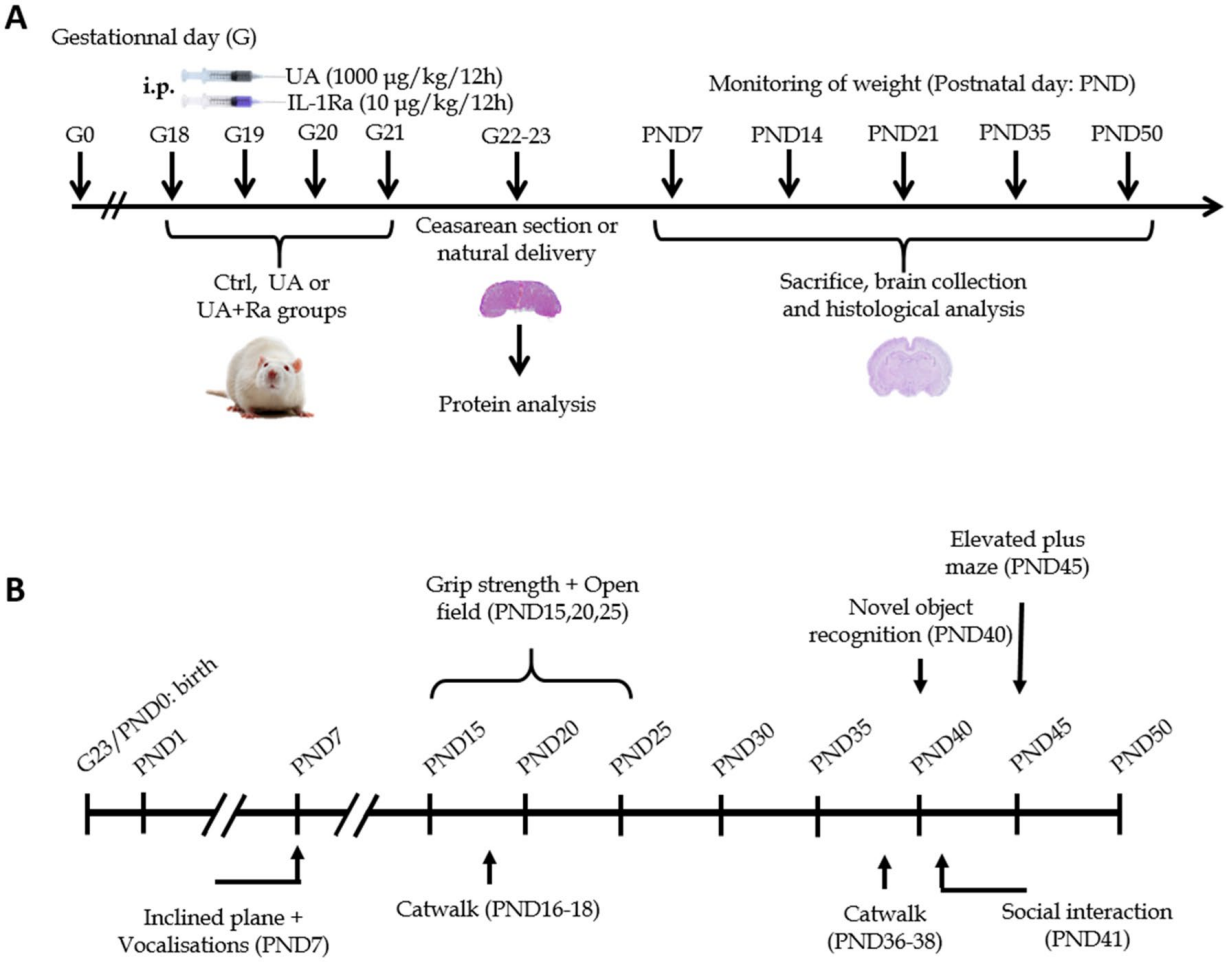
Experimental schedule of the model. Pregnant dams were injected i.p. every 12 h with uric acid from gestational day (G)18 to 21. C-section were performed at G22 for placental protein analysis. Natural delivery also occurred in parallel to evaluate the pups in the postnatal period from postnatal day (PND)1 through PND50 (**A**). Behavioural assessment schedule of the model. Eight different behavior tests were performed from postnatal day (PND) 1 to PND50 to evaluate motricity and cognitive functions (**B**).

We used our published model of prenatal inflammation of non-pathogenic origin^33^. Briefly, timed mated Sprague–Dawley rats were obtained at gestational day (GD) 13 (Charles River Laboratories, QC, Canada) and kept in a controlled 20 °C environment with a 12 h light/dark cycle and access to food and water ad libitum. Pregnant dams were injected intraperitoneally every 12 h starting at GD18 until GD21 with uric acid crystals (UA; monosodium urate; 1000 µg/kg/12 h; Sigma-Aldrich) combined with potassium oxonate (125 mg/kg/day; Sigma-Aldrich), an inhibitor of the enzyme uricase which is absent in humans but present in rodents and rapidly degrades uric acid or with PBS as our control group^57^. Rapidly after these injections, rats were treated either with the recombinant human IL-1Ra (10 mg/kg/12 h, Kineret, Sobi-Swedish Orphan Biovitrum, ON, Canada) or vehicle (PBS). Caesarean-section was performed at GD22 and placentas rapidly collected, snap-frozen and stored at − 80 °C for future analysis (see section below). Other dams delivered naturally, and the pups were assessed daily from postnatal day (PND) 1 to PND50 for weight gain and developmental milestones (e.g. eye opening, ears unfolding and fur appearance) (see Fig. 7A for experimental design). Immunohistological analysis of the postnatal brain as well as behavioral assessment were performed (see sections below for details).

No differences between vehicle (PBS), potassium oxonate or IL-1Ra only treated groups were observed in term of inflammatory markers in the placenta or in any of the postnatal analysis and all were combined and are referred to as control (Ctrl) from now on. For the postnatal weight analysis, N = 6–10 litters (i.e. PBS = 8; IL-1Ra = 6; UA = 10; UA + Ra = 6) at PND7/21 and at PND35/50, N = 6 litters each groups were used (1 male and 1 female per litters included in each experimental group).

### Protein extraction and analysis

Proteins were extracted as previously described^33^. Briefly, placentas were homogenized in lysis buffer containing 1% Triton X-100 (Sigma-Aldrich, ON, Canada) and protease inhibitor cocktail (Calbiochem, Millipore, ON, Canada), and centrifuged at 13,000 rpm for 10 min at 4 °C. Supernatants were collected and stored at − 20 °C until analysis. Protein concentration was determined using the Bradford assay (Bio-Rad, ON, Canada). Levels of cytokines (IL-1β, IL-6 and TNF-α) were determined by ELISAs (Duoset, R&D Systems) following manufacturer’s instructions. A N of 6–12 dams (i.e. Ctrl = 12; UA = 12; UA + Ra = 6) and 3 randomly selected placenta/dams were used.

### Histological analysis

Five um-thick sections of paraffin-embedded brains were obtained and processed for H&E staining and immunohistochemistry as previously described^33^. The impact of in-utero exposure to inflammation was determined at 4 postnatal brain developmental stages based on previous studies^58, 59^. These were postnatal day (PND) 7 to assess the developmental equivalent of the infant human brain at term (36–40 weeks gestation); PND21 as the early adolescence developmental stage; PND35 as the early adult stage and PND50 for the adult brain. Both white and grey matters were evaluated at each timepoint (i.e. corpus callosum—CC; cingulum—CG; cortex; hippocampal-dentate gyrus—DG; hippocampal-cornus amonis—CA; and internal capsule—IC). The impact of sterile inflammation on the developing brain was evaluated using the following antibodies: ionized calcium binding adapter molecule 1 (Iba1; Wako Chemicals, VA, USA), glial fibrillary acid protein (GFAP; Chemicon, ON, Canada), myelin basic protein (MBP; Chemicon, ON, Canada), doublecortin (DCX; Abcam, MA, USA) and neuronal nuclei (NeuN; Millipore, MA, USA). Matched secondary HRP-conjugated antibodies were used, either anti-rabbit-HRP, or anti-mouse-HRP (BioRad, ON, Canada), and revealed using 3, 3-diaminobenzidine (DAB; VWR, ON, Canada). Sections were counterstained with hematoxylin. Images were obtained with a slide scanner (Axioscan; Zeiss, ON, Canada), analysis performed using ImageJ (NIH Image) and cell counting was done by a blinded individual. For these experiments, N = 8–16 litters (i.e. PBS = 11; IL-1Ra = 8; UA = 16; UA + Ra = 12) were used with 1 male and 1 female per litters included in each experimental group.

### Behavioral assessment

Several behavioral tests (e.g., inclined plane, ultrasound vocalisation, grip strength, open field, catwalk, social interaction, novel object recognition, elevated plus maze) were performed in the postnatal period (see Fig. 7B for the testing schedule). A period of 5 days of rest (i.e. no testing) was used between different tests. The animals were acclimatized into the room for at least 30 min before any behavioural assessment. 6 litters, with 1 male and 1 female pup each, were analysed in each group.

*Inclined plane* was performed at PND7 to evaluate motor reflex as previously described^60^. Pups were placed head down on a grid plane at 45 degrees. The time for the animal to reach a full head up position was measured.

*Ultrasound vocalisations* were analysed to assess pups’ communications as previously described^4^. At PND7, the pups were separated from their mother/littermate and placed in a box with ultrasound caption. The vocalisations (frequencies: 30–65 kHz) were measured for 3 min using the Avisoft SASLab software (Avisoft Bioacoustics, Germany).

*Grip strength* was measured using a grip strength apparatus (BIOSEB, USA) on PND15, 20 and 25 as previously reported^16^. Briefly, the front paws were placed on the grid and the tail pull back gently until the pup let go. The maximal force used by the pup was recorded.

*Open field (OF)* was used at PND15, PND20 and PND25 to assess spontaneous locomotor activities and exploratory behavior as previously described^37^. Rats were always placed facing the same direction in the upper right corner of the OF apparatus (40 × 40 × 40 cm Plexiglas box). Rat spontaneous movements were recorded for 5 min with a camera placed directly over the apparatus (ANY-maze video-tracking system, Stoelting Co., USA). Recording began as soon as the animal was placed in the OF. The total distance traveled, line crossing, mean speed and the mobile/immobile time were analysed.

*Catwalk* was used to evaluate the gait of the animal by analysing several parameters such as, speed, each paw pressure, coordination, etc. as previously described^61^. This test was performed daily from PND16 to 18 and from PND36 to 38. The animal was placed in a glass corridor and a camera underneath recorded gait. The results were recorded and analysed using the CatWalk XT 10.6 software (Noldus, USA).

*Novel object* test is used to evaluate short term memory as previously described^62^. On PND38, the animal was placed in a plexiglass box (same as the OF apparatus, see above) for 5 min habituation. On the next day, the animal was placed in the box with two identical objects for a 5 min exploration period. Finally, on PND40, the animal was placed again into the box but one of the objects was replaced by a new one. The time the animal took to explore each of the two objects was recorded within a period of 5 min. The recording was performed using a camera above the apparatus (ANY-maze video-tracking system, Stoelting Co., USA) which tracked the animal interactions with the objects.

*Social interaction* pups were placed in a box (same apparatus as the OF) with a stranger rat (same sex but from a different litter) and the interactions (duration and type of interaction) between the two animals for 5 min were recorded by an overhead camera (ANY-maze video-tracking system, Stoelting Co., USA) and manually scored by an observer blinded to the animal experimental conditions, as previously described^4^.

*Elevated plus maze* at PND45, anxiety was measured using a plus (+) shape platform elevated 96 cm above the ground with two open and two closed arms as previously described^16^. The animal can therefore choose a secluded (if more anxious) or open (if more exploratory) environment. The animal was placed in the middle of the apparatus and their movements recorded for 5 min with a camera above the apparatus and the animal trajectory was tracked and analysed (ANY-maze video-tracking system, Stoelting Co., USA).

### Statistical analysis

Data are presented as mean ± standard error of the mean (SEM). Data were analyzed using one-way ANOVA with Tukey’s multiple comparisons or Fisher’s exact test, as appropriate. All analysis were made on male and female separately and, since no differences were found in any of the parameters studied, they were combined. Statistical analysis was performed with GraphPad Prism 9.2.0 (GraphPad Software, CA; www.graphpad.com) and a p value of < 0.05 was considered statistically significant.

## Supplementary Information

Supplementary Figure S1.
