# Supplementary material for: Prenatal administration of IL-1Ra attenuate the neurodevelopmental impacts following non-pathogenic inflammation during pregnancy
Source: Sci Rep. 2021 Dec 3;11:23404. doi: 10.1038/s41598-021-02927-3 (PMC8642433; doi:10.1038/s41598-021-02927-3)
Supplement: Supplementary file 1 — Supplementary Figure S1. [file 41598_2021_2927_MOESM1_ESM.docx]

**
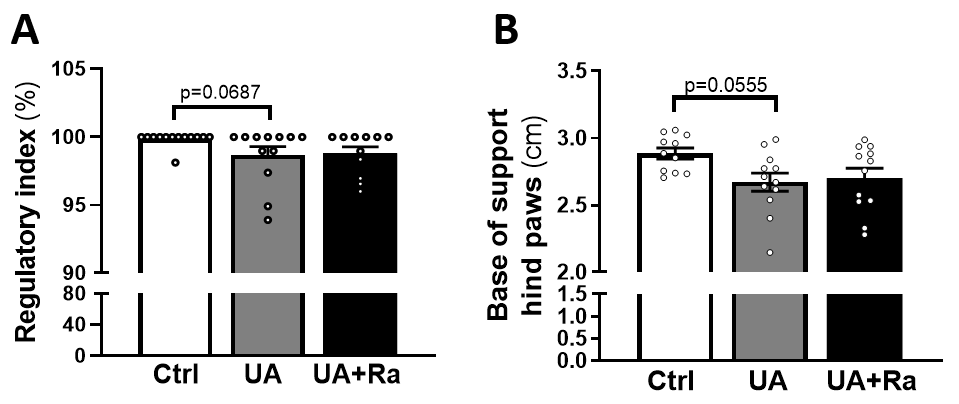
**

**Supplementary Figure S1. Tendency of impaired motricity at PND16.** Pups expose to uric acid has a tendency of decreased regulatory index (C) and base of support of the hind paws (D) as evaluated by the catwalk. N=6 litters/group. Results presented as mean ± SEM. Statistical analysis by one-way ANOVA with Tukey’s multiple comparisons test with GraphPad Prism 9.2.0 (GraphPad Software, CA); www.graphpad.com.
